# Public Image of Nursing Among High School Adolescents in Türkiye: Implications for the Future Healthcare Workforce

**DOI:** 10.3390/healthcare14111483

**Published:** 2026-05-27

**Authors:** Filiz Coşkun, Derya Gündüz Hoşgör, Ece Uysal Kasap, Tuba Çatak

**Affiliations:** 1 Hamidiye Vocational School of Health Services, University of Health Sciences, Tıbbiye Street No. 38, 34668 Istanbul, Türkiye; 2 Uşak University Medical Faculty Training and Research Hospital, Fevzi Çakmak District Denizli Street No. 4, 64100 Uşak, Türkiye; 3 Arnavutköy Darülaceze Vocational School of Health Services, University of Health Sciences, Eski Edirne Asfaltı No. 1903, 34277 Istanbul, Türkiye; 4 Nursing Department, Faculty of Health Sciences, Istanbul Gedik University, Ankara Street No. 280, 34906 Istanbul, Türkiye

**Keywords:** healthcare workforce, nursing image, high school adolescents, career intention, nursing profession

## Abstract

**Background:** The global shortage of healthcare workers continues to grow each year. In particular, low nurse staffing levels are known to be associated with adverse patient outcomes. Helping adolescents understand the nursing profession beyond stereotypical societal perceptions—and recognize its full range of roles—may make nursing a more attractive career option. However, little is known about the perceptions of adolescents who are in the process of making career choices. **Objective:** This study aims to determine the perceptions of high school adolescents in Türkiye regarding the image of the nursing profession. **Method:** The sample of this cross-sectional study consisted of 581 high school adolescents in Türkiye. Data were collected using the Descriptive Characteristics Form and the Adolescents’ Perceptions of the Image of Nursing Scale (APNIS). In addition to descriptive statistics, independent-samples *t*-tests, one-way ANOVA, and Chi-Square analyses were performed. **Results:** The findings indicated that adolescents generally hold a positive perception of the nursing profession, with higher scores in the professional image, perception, and care and therapeutic role subscales, and lower scores in the communication, informative role, and healing environment subscales. A total of 65.7% of the adolescents reported that they did not intend to choose nursing as a career. The intention to choose nursing as a career was higher among adolescents who did not have a nurse in their family (*p* < 0.05). **Conclusions:** The results indicate that positive perceptions of the nursing image alone may not be sufficient in adolescents’ career decision-making and underscore the importance of presenting the profession’s roles in a comprehensive and realistic manner. While adolescents with a nurse in the family demonstrated more positive perceptions of the nursing profession (higher APNIS scores), their intention to choose nursing as a career was lower compared to those without a nurse in the family. These findings suggest that familiarity with the profession may positively influence professional image while simultaneously reducing career intention, possibly due to greater awareness of occupational challenges. The findings provide important insights into how adolescents form their perceptions of the image of nursing and may contribute to future research and educational initiatives aimed at increasing interest in the nursing profession.

## 1. Introduction

The Global Strategy on Human Resources for Health: Workforce 2030 estimates that the global shortage of health workers could reach 18 million by 2030 [1]. Similarly, the International Council of Nurses estimates that approximately 13 million additional nurses will be needed over the next decade [2]. This issue is not limited to local or national contexts; rather, it represents a global concern [3]. Evidence indicates that inadequate nurse staffing levels are associated with adverse outcomes, including increased mortality rates [4,5].

In today’s increasingly globalized healthcare environment, rapid technological advances, infectious disease outbreaks, and ongoing pandemic threats have made nursing roles broader and more complex than ever before [6]. Despite the widespread recognition nurses received during the recent pandemic, numerous studies have shown that public understanding of the profession remains limited and often fails to reflect the diverse leadership roles nurses undertake in education, clinical practice, policy development, research, management, and public health initiatives [7,8,9]. How nursing is perceived by society—its public image—has been an ongoing topic of global discussion [10]. Although perceptions of the profession have evolved over time, a societal image that fully recognizes nursing’s scientific and professional development has yet to be established [10,11]. A lack of awareness regarding the academic, scientific, and professional status of nursing persists not only in developing countries such as China but also in developed countries, including Spain and the United States [9,12,13]. This risks diminishing the perceived impact of the profession and may make nursing a less attractive career option for those considering entering the field [14,15].

Adolescence (ages 10–19) represents a critical developmental stage during which individuals begin to understand the social barriers that may influence their choice of profession and their ability to pursue a suitable career [16,17]. Adolescents’ perceptions of the nursing profession tend to reproduce its historical image, portraying nurses as kind, self-sacrificing, and compassionate women [18]. However, fundamental aspects of the profession—such as its autonomy, multifaceted roles, evidence-based care processes, nursing practices, leadership within the healthcare system, and academic career opportunities—remain largely underrecognized [17,19]. A study conducted among high school adolescents in the United States and Israel reported that nursing is not considered an ideal profession and that nurses are generally perceived as overworked individuals with limited opportunities for leadership and autonomy [20]. Similarly, research conducted in Scotland indicated that the vast majority of high school adolescents would never consider choosing nursing as a career, while only a small proportion reported that they might consider it in the future [21]. Findings from a general population sample in Italy showed that the lowest perception scores regarding the image of nursing were observed among high school adolescents [22]. Understanding how high school adolescents—who are at a critical stage of career decision-making—perceive the image of nursing, and identifying and addressing factors that negatively influence these perceptions, may help increase interest in the nursing profession [23].

In Türkiye, career choice typically occurs around the age of 18, following the completion of high school, through the national university entrance examination. This process coincides with adolescence, a period characterized by identity exploration and rapid biopsychosocial changes [24,25]. A review of previous studies conducted in Türkiye shows that most were carried out within a single province (e.g., Sinop, Çankırı, Istanbul). Some studies used researcher-developed questionnaires [26,27], while others employed validated instruments applicable to the general population, such as nursing image scales [28,29]. Overall, these studies indicate that although high school adolescents in Türkiye hold partially positive perceptions of nursing, they generally do not consider it as a career option, and their awareness of the profession’s educational requirements, knowledge base, skills, and professional roles remains limited. Furthermore, the measurement tools used in existing studies are not specific to adolescents and have been applied within limited geographical regions. These limitations limit the ability to obtain comprehensive, generalizable findings regarding high school adolescents’ perceptions of the nursing profession. Therefore, there is a clear need for studies conducted with samples including high school adolescents from multiple provinces in Türkiye, using psychometrically robust instruments specifically developed for the adolescent population.

This study addresses this gap by reaching high school adolescents from various regions of Türkiye through online communication tools and by using a valid and reliable instrument specifically developed to measure adolescents’ perceptions of the nursing image. In doing so, the study seeks to generate more generalizable findings regarding adolescents’ perceptions of nursing and to propose practical solutions to address the growing shortage of nurses.

The aim was to determine the perceptions of high school adolescents in Türkiye regarding the image of the nursing profession.

The research questions included the following:

(1) What are high school adolescents’ perceptions of the image of nursing?
(2) Which sociodemographic characteristics influence high school adolescents’ perceptions of the image of nursing?
(3) What are high school adolescents’ intentions regarding choosing nursing as a career?

## 2. Methods

### 2.1. Study Design

This was a descriptive cross-sectional study to determine the image of nursing as perceived by adolescents living in Türkiye and to identify factors associated with these perceptions.

### 2.2. Population and Sample

The population of the study consisted of 5,328,862 high school adolescents enrolled in public and private high schools under the Turkish Ministry of National Education in the 2025–2026 academic year [30]. Given the large size of the population and its wide distribution across Türkiye, a non-probability sampling technique, namely convenience sampling, was employed. Participation in the study was based on voluntary consent, and adolescents enrolled in public and private high schools that were accessible to the researchers were included in the sample. The required sample size was calculated using a 95% confidence level and a 5% margin of error. The calculation indicated that a minimum of 384 adolescents would be sufficient. Under the specified assumptions, a minimum sample size of 384 adolescents was considered sufficient to provide adequate statistical power [31].

Parameters:

- N = 5,328,862;
- T = 1.96 (95% confidence level);
- *p* = 0.50 (assumption of maximum variance);
- q = 0.50;
- d = 0.05.

Estimation:

*n* ≈ 384.

The sample size was exceeded to increase the statistical power of the study, minimize sampling error, and improve the robustness and generalizability of the findings. Due to the nature of the online convenience sampling method and indirect distribution of the survey link through parents, the exact number of individuals who received the invitation could not be determined. Therefore, a precise response rate could not be calculated.

Inclusion criteria:

The study included adolescents aged 14–18 years who were enrolled in public and private high schools affiliated with the Ministry of National Education during the 2025–2026 academic year. Since nursing education in Türkiye is provided at the undergraduate level in universities, the adolescents had not received any formal education in nursing. Adolescents were eligible if they volunteered to participate, had parental consent, provided informed assent, and completed the data collection instruments fully.

Exclusion criteria:

Adolescents who declined to participate, those who submitted incomplete or improperly completed questionnaires, individuals enrolled in educational levels outside the scope of the study (e.g., middle school), and those under 14 or over 18 years of age were excluded from the analysis.

### 2.3. Data Collection Instruments

Data were collected using a Descriptive Characteristics Form developed by the researchers and the Adolescents’ Perceptions of the Nursing Image Scale (APNIS).

#### 2.3.1. Descriptive Characteristics Form

The form consisted of 12 items selected based on variables commonly used in the literature to describe adolescents’ characteristics. These variables included age, gender, school type, history of hospitalization, and intention to choose nursing as a career, among others.

#### 2.3.2. The Adolescents’ Perceptions of the Nursing Image Scale (APNIS)

The scale was developed and validated by Cırık, Aksoy, and Bektaş. This scale was developed to assess perceptions of the nursing image among adolescents aged 12 to 18 years [23]. It consists of 38 items, each rated on a five-point Likert scale (1 = strongly disagree, 2 = disagree, 3 = undecided, 4 = agree, 5 = strongly agree). The APNIS scale has six subscales. These subscales are: Professional image (items 1–10), Perception (items 11–19), Care and therapeutic role (items 20–27), Communication (items 28–31), Informative role (items 32–35), and Healing environment (items 36–38). There are no reverse-coded items in the scale.

The minimum possible score on the scale is 38 (38 × 1), and the maximum is 190 (38 × 5). Higher scores indicate more positive perceptions of the nursing image among adolescents. Based on the scores obtained from the scale, adolescents’ perceptions of the nursing image decrease as scores approach 38 and, conversely, increase as scores approach 190. The Cronbach’s alpha reliability coefficient for the scale was reported to range from 0.70 to 0.90.

### 2.4. Data Collection

Data were collected using an online questionnaire administered via Google Forms. The researchers did not directly access or obtain parents’ personal contact information from educational institutions or official databases. As part of the convenience sampling procedure, the survey link was shared with accessible parent groups via commonly used mobile messaging platforms. Participation was entirely voluntary, and no personal contact information was collected, accessed, or stored by the researchers at any stage of the study. The parents were asked to share the link with their adolescent children. To ensure that responses reflected the adolescents’ own views, clear instructions were provided at the beginning of the questionnaire, emphasizing that the survey should be completed independently by the adolescents without parental assistance or influence. To minimize duplicate responses, the questionnaire settings allowed only one response per device/account. Additionally, participating adolescents were assured of anonymity and confidentiality in order to minimize social desirability bias and response distortion.

Following ethics committee approval from the Istanbul Gedik University Health Sciences Ethics Committee on 4 February 2026, data were collected during the spring semester of the 2025–2026 academic year (5 February–5 March 2026). Participation was entirely voluntary, and no identifying information was collected.

Given that the study population consisted of minors, parental consent was obtained prior to participation. Adolescents were also required to provide their own informed assent before proceeding with the questionnaire. The consent procedure was conducted in accordance with national ethical and legal regulations governing research involving individuals under the age of 18. An informed consent statement was presented to parents at the beginning of the survey, outlining the purpose of the study, the voluntary nature of participation, and data confidentiality. Only adolescents whose parents provided consent were allowed to proceed to the questionnaire.

Despite these precautions, the possibility of parental influence on responses cannot be entirely ruled out and should be considered as a potential source of bias.

### 2.5. Data Analysis

Data were analyzed using IBM SPSS Statistics for Windows, version 27.0 (IBM Corp., Armonk, NY, USA). Adolescents’ characteristics were evaluated using descriptive statistics (number, percentage, frequency, standard deviation, minimum, and maximum values). For reliability, Cronbach’s alpha coefficients were calculated to assess internal consistency. The normality assumption was evaluated based on skewness and kurtosis values. The ±2 threshold, as recommended by Tabachnick, Fidell, and Ullman (2013), was used for this assessment [32].

Since the normality assumption was met, independent-samples *t*-tests and one-way ANOVA were used to compare APNIS total and subscale scores across adolescents’ characteristics. The Chi-square test was used to examine the relationship between adolescents’ characteristics and their intention to choose nursing as a career. Statistical significance was accepted at the 95% confidence level (*p* < 0.05).

### 2.6. Ethics Approval

Ethical approval was obtained from the Istanbul Gedik University Health Sciences Ethics Committee before the study was initiated (Decision No. E-11470191-050.04-2026.173340.2, dated 4 February 2026) (Supplementary Materials File S1). The study was conducted in accordance with the principles of the Declaration of Helsinki.

Informed Consent Statement: Informed consent was obtained electronically from the parents or legal guardians of all participating adolescents prior to data collection. Parents were informed about the purpose of the study and voluntarily approved their child’s participation through the Google Forms system before accessing the questionnaire.

## 3. Results

Among the adolescents participating in the study, 23.9% were 15 years old, 53.2% were female, 30.1% were in the 9th grade, and 90.2% attended public schools. Regarding parental education, 27.5% of adolescents’ mothers held a bachelor’s degree, whereas 17.7% of adolescents’ fathers had completed primary school. Most adolescents (88.3%) reported having no chronic illness. More than half of the adolescents (55.8%) had never been hospitalized, while 72.5% reported that at least one family member had previously been hospitalized.

In addition, 23.6% of adolescents reported having a nurse in their family, and 31.8% reported obtaining information about the nursing profession from family members or close relatives. Overall, 65.7% of the adolescents reported that they did not intend to choose nursing as a career (Table 1).

The mean APNIS score among the adolescents was 153.3 ± 21.54. When the subscales were examined, the mean scores were 42.62 ± 5.19 for professional image, 33.97 ± 6.96 for perception, 34.06 ± 4.59 for care and therapeutic role, 16.31 ± 2.89 for communication, 14.15 ± 3.39 for informative role, and 11.91 ± 2.26 for healing environment (Table 2).

When APNIS scores were analyzed, several patterns emerged. Male adolescents scored significantly higher on the perception subscale. School type was also associated with differences in scores: adolescents attending public schools had higher scores on the communication subscale. Differences were also observed according to the presence of a nurse in the family. Adolescents with a nurse in the family reported higher scores on professional image, communication, informative role, healing environment, and overall APNIS scores (*p* < 0.05) (Table 3).

Statistically significant relationships were found between adolescents’ characteristics and their intention to choose nursing as a career (*p* < 0.05). Male adolescents reported a higher intention to choose nursing as a career compared with female adolescents. Adolescents attending private schools had higher intention levels than those in public schools. Adolescents without a nurse in the family demonstrated a higher intention to choose nursing as a career. Parental education levels were also associated with intention; higher mother’s education was generally linked to increased intention, whereas father’s education showed a non-linear pattern. Additionally, the source of information about nursing was significantly related to intention, with higher intention observed among adolescents who reported television series/films as their source of information about nursing (Table 4).

The overall APNIS demonstrated excellent internal consistency, with a Cronbach’s alpha of 0.96. Subscale reliability coefficients ranged from 0.73 (Healing Environment) to 0.94 (Perception), indicating good internal consistency across all dimensions. Presenting these values separately allows for a clearer interpretation of each subscale’s measurement reliability (Table 5).

## 4. Discussion

This cross-sectional study examined the perceptions of high school adolescents in Türkiye regarding the image of the nursing profession. A total of 581 high school adolescents were included, and their perceptions of nursing were analyzed.

The findings indicate that adolescents generally hold a positive image of nursing. The mean APNIS score suggests that the perceived image of nursing among adolescents was at a good level. Examination of the subscales revealed that the professional image dimension had the highest mean score, followed by relatively high scores in the care and therapeutic role dimension. In contrast, the healing environment dimension had the lowest mean score. These findings suggest that adolescents tend to perceive nursing primarily in terms of direct care and therapeutic roles, likely reflecting the dominance of care-oriented representations in social and media contexts, as well as limited exposure to its multidimensional professional roles.

A considerable portion of studies examining the image of nursing have been conducted among nurses and nursing students [33,34]. In contrast, community-based studies, particularly those involving adolescents, remain relatively limited. This highlights the importance of research exploring how perceptions of nursing develop during earlier stages of life.

Previous studies have reported that public perceptions of nursing vary across countries and contexts. Studies conducted in Türkiye have reported the image of nursing to be moderately positive [35] or relatively weak [36]. Similarly, studies from countries such as Iran and Argentina have indicated that the public image of nursing is perceived negatively or at a low level [37,38]. In contrast, several studies conducted during and after the COVID-19 pandemic reported a more positive perception of nursing, largely attributed to the increased visibility of nurses during the pandemic [39,40,41]. These findings suggest that the public image of nursing may change depending on social and contextual conditions. Indeed, international literature emphasizes that the image of nursing is not a fixed construct but rather shaped by multiple factors such as societal perceptions, media representations, and structural characteristics of healthcare systems [10,42]. In this context, the relatively positive image observed in the present study may partly be associated with the increased visibility of nursing in the post-pandemic period.

The results also showed that scores for the care and therapeutic role dimension were relatively high, whereas scores for the healing environment and informative role dimensions were comparatively lower. Cırık et al. [43] reported that children and parents often describe nurses using passive and service-oriented metaphors, and that children tend to hold idealized images of the profession. Additionally, adolescents sometimes interpret nurses’ roles as supportive, associated with physicians [18]. These findings support the interpretation that adolescents predominantly perceive nursing through the lens of direct patient care and treatment-related responsibilities. Although nursing is inherently a care-focused profession, this pattern suggests that adolescents may not fully recognize the profession’s broader and multidimensional professional roles.

The majority of adolescents in this study were female. The analysis showed no statistically significant difference in total scale scores according to gender. Similarly, several studies have reported no significant relationship between gender and perceptions of the nursing image [44,45]. However, a study by Baykara Mat and Baykal [35] found that gender may influence perceptions of nursing.

In the present study, male adolescents demonstrated higher scores on the perception subscale, and gender differences were also observed in the intention to choose nursing as a career. Historically, nursing has often been positioned as a female-dominated profession, and gender has played an important role in shaping the professional image of nursing (10). Previous research has shown that adolescents frequently describe nursing as a care-oriented profession associated with women [18]. Nevertheless, studies examining male adolescents’ motivations for choosing nursing have identified career opportunities, professional development expectations, and job security as important motivating factors [46,47]. The higher perception scores and stronger intention to choose nursing as a career observed among male adolescents in this study may suggest that some male adolescents evaluate the profession more positively, and that positive perceptions of the profession may support career interest.

The majority of adolescents reported that they did not intend to choose nursing as a career. This finding is consistent with several previous studies reported in the literature. Research suggests that although the professional image of nursing is an important factor influencing career choice, career decisions are also shaped by various individual and environmental factors, including social status, career expectations, and personal motivations [29,48]. This finding highlights a notable discrepancy between a generally positive image of nursing and a relatively low willingness to choose nursing as a career, which may be explained by the influence of multiple individual and contextual factors such as career expectations, perceived social status, and limited awareness of the broader professional roles of nursing.

A noteworthy finding is that many adolescents who had a nurse in their family still reported that they did not intend to choose nursing as a career, a result that is consistent with findings from some previous studies [48,49]. Similarly, Koç and Sağlam [26] reported that although high school adolescents perceived nursing as an important profession, they did not necessarily consider choosing it as a career. Adolescents with a nurse in their family had higher scores on professional image and related subscales, including communication, the informative role, and the healing environment. This may be associated with greater exposure to and observation of the profession. However, it is particularly notable that adolescents without a nurse in their family reported a higher intention to choose nursing. This discrepancy may be explained by the fact that close exposure to the profession may facilitate a more realistic understanding of its roles, while also increasing awareness of its challenges.

Previous research suggests that adolescents often associate nursing primarily with helping and caring roles and may interpret the nurse’s role as supportive to physicians [18,50]. Furthermore, adolescents’ perceptions of nursing may be influenced by family members, peers, and other social environments, which can shape their educational and career preferences [18]. Although nursing is widely recognized as a trusted and respected profession, ongoing debates remain regarding its public image and career attractiveness [10]. In this context, adolescents with a nurse in their family may have greater awareness of the profession’s working conditions, whereas those without direct exposure may evaluate nursing within a more limited or idealized framework. These findings suggest that close exposure to the profession may strengthen professional image but does not necessarily increase career intention.

Although the visibility of nursing in the media has increased in Türkiye in the post-pandemic period, it has often been framed through narratives of heroism or self-sacrifice and has not fully reflected the multidimensional nature of the professional role [51]. While this factor was not directly measured in the present study, it may partly explain why adolescents evaluate the nursing profession positively while not fully recognizing its professional dimensions. International comparisons indicate that the rate at which adolescents choose nursing as a career varies across countries, and that structural factors such as economic conditions, public investment in health systems, and the safety of the working environment play a determining role in these preferences [52]. Despite the academic development of nursing in Türkiye, the perception that working conditions and system-level incentives are not sufficiently attractive for adolescents may have limited the rate of preference for the profession.

The analysis based on sources of information about nursing showed that the intention to choose nursing as a career differed significantly across groups. Adolescents who reported obtaining information from television series and films demonstrated relatively higher intention scores. However, no significant differences were found in overall nursing image scores according to information sources. Previous studies have emphasized that the image of nursing can be influenced by media representations [42]. While the increased visibility of nurses in the media after the pandemic may have contributed to positive perceptions [51], a study from Iran has reported that portrayals of nurses in television programs and films may sometimes be negatively perceived by both nurses and the public [41]. These findings suggest that media representations may exert complex and multidimensional influences on perceptions of nursing and career intentions.

Finally, communication subscale scores were higher among adolescents attending public schools, suggesting that the educational environment may influence perceptions of nurses’ communication roles. Additionally, the intention to choose nursing as a career increased as maternal education level increased, whereas the opposite pattern was observed for paternal education level. These findings indicate that family background and educational environment may influence adolescents’ professional perceptions and career orientations. Previous research similarly emphasizes that perceptions of nursing among adolescents are shaped by multiple social influences, including family, educational contexts, and media exposure [10,18,52].

Overall, the findings suggest that perceptions of nursing begin to develop at an early age, highlighting the importance of initiatives aimed at helping adolescents better understand the professional roles and responsibilities of nurses, and the need to frame these perceptions within a more comprehensive and professionally grounded perspective.

In addition to these findings, several practical implications emerge. Variations in adolescents’ perceptions of nursing indicate that both early contact with the profession and educational experiences play a significant role in shaping its professional image. School-based activities, opportunities to observe diverse professional roles, and more accurate portrayals of nursing in educational and media settings may foster a more comprehensive understanding of the profession among adolescents. These findings may inform educational initiatives aimed at improving adolescents’ understanding of nursing and its multifaceted professional roles.

## 5. Limitations

The findings of this study should be interpreted considering several limitations. First, the use of a convenience sampling method and online data collection may limit the generalizability of the findings to all adolescents in Türkiye. Therefore, the findings should be interpreted with caution in terms of national representativeness. Second, because the questionnaire was administered online through parents, the possibility of parental influence on adolescents’ responses cannot be entirely excluded, despite the instructions emphasizing independent completion. Third, all data were based on self-report measures and may therefore be subject to response and social desirability biases. In addition, due to the distribution method used, an exact response rate could not be calculated. Finally, the cross-sectional design of the study does not allow causal inferences regarding the relationships between variables.

## 6. Conclusions

The findings of this study indicate that adolescents generally hold positive perceptions of the nursing image; however, most do not intend to choose nursing as a career. Analysis of the scale subdimensions shows that adolescents tend to perceive nursing primarily in terms of direct care and therapeutic roles, while their perceptions of communication, informative roles, and healing environment roles remain limited. These findings highlight the need to broaden adolescents’ traditional perceptions of nursing and to promote awareness of its multifaceted professional roles, including education, practice, research, management, and leadership.

While adolescents with a nurse in the family demonstrated more positive perceptions of the nursing profession (higher APNIS scores), their intention to choose nursing as a career was lower compared to those without a nurse in the family. These findings suggest that familiarity with the profession may positively influence professional image while simultaneously reducing career intention, possibly due to greater awareness of occupational challenges.

In conclusion, this study demonstrates that positive perceptions of the nursing image alone may not be sufficient in adolescents’ career decision-making and underscores the importance of presenting the profession in a comprehensive and realistic manner. The findings provide important insights into how high school adolescents—who are at a critical stage of career choice—form their perceptions of nursing, and may inform future research and educational initiatives aimed at increasing interest in the profession.

## Figures and Tables

**Table 1:** Adolescents’ Descriptive Characteristics.

Variables		*n*	%	Variables		*n*	%
Age	14 years	95	16.4	Grade level	9th grade	175	30.1
	15 years	139	23.9		10th grade	148	25.5
	16 years	131	22.5		11th grade	93	16.0
	17 years	102	17.6		12th grade	165	28.4
	18 years	114	19.6				
Gender	Female	309	53.2	Presence of a chronic disease	Yes	68	11.7
	Male	272	46.8		No	513	88.3
School type	Public school	524	90.2	History of hospitalization	Yes	257	44.2
	Private school	57	09.8		No	324	55.8
Mother’s education level	Illiterate	22	03.8	Father’s education level	Illiterate	6	01.0
	Primary school	124	21.3		Primary school	163	17.7
	Secondary school	71	12.2		Secondary school	100	17.2
	High school	149	25.6		High school	144	24.8
	Bachelor’s degree	160	27.5		Bachelor’s degree	153	26.3
	Postgraduate	33	05.7		Postgraduate	57	09.8
	Unknown	22	03.8		Unknown	18	03.1
Family member previously hospitalized	Yes	421	72.5	Nurse in the family	Yes	137	23.6
	No	160	27.5		No	444	76.4
Source of information about nursing	TV series/films	99	17.0	Intention to choose nursing as a career	Yes	199	34.3
	Social media	63	10.8		No	382	65.7
	School/teachers	63	10.8				
	Family/close relatives	185	31.8				
	Hospital experience	123	21.2				
	Internet/news websites	48	08.3				
Total		581	100.0	Total		581	100

**Table 2:** Mean APNIS Scores and Subscale Scores of the Adolescents.

APNIS and Subscales	Mean	SD	Min.	Max.
Professional image	42.62	5.19	10	50
Perception	33.97	6.96	9	45
Care and therapeutic role	34.06	4.59	8	40
Communication	16.31	2.89	4	20
Informative role	14.15	3.39	4	20
Healing environment	11.91	2.26	3	15
Total scale	153.3	21.54	38	190

Abbreviation: SD, standard deviation; min.: minimum; max.: maximum.

**Table 3:** Comparison of APNIS and Subscale Scores by Adolescents’ Characteristics.

Variables		Professional Image	Perception	Care & Therapeutic Role	Communication	Informative Role	Healing Environment	Total Scale
Gender	t	−0.402	−2.034	−0.722	−1.076	−1.123	−1.392	−1.377
	*p*	0.688	**0.040**	0.470	0.283	0.262	0.164	0.169
School type	t	1.404	1.556	1.457	2.189	0.787	1.377	1.738
	*p*	0.164	0.124	0.149	**0.032**	0.434	0.173	0.088
Having a Nurse in the Family	t	2.506	1.556	1.494	2.351	2.579	2.330	2.432
	*p*	**0.013**	0.121	0.136	**0.020**	**0.011**	**0.021**	**0.016**
Mother’s education level	F	1.706	0.875	1.614	1.778	1.456	0.508	1.225
	*p*	0.117	0.513	0.141	0.101	0.191	0.803	0.291
Father’s education level	F	0.432	0.555	0.390	0.713	0.417	0.737	0.456
	*p*	0.858	0.776	0.885	0.639	0.868	0.620	0.841
Source of information about nursing	F	0.647	0.363	1.634	0.346	0.299	0.487	0.382
	*p*	0.664	0.874	0.149	0.885	0.914	0.786	0.860

t = independent-samples *t*-test; F = one-way ANOVA; *p* < 0.05 was considered statistically significant (in bold).

**Table 4:** Relationship Between Adolescents’ Characteristics and Intention to Choose Nursing as a Career.

Variables	Intention to Choose Nursing as a Career		X^2^	*p*
**Gender**	**Yes (%)**	**No (%)**		
Female	28.3	71.7	8.020	0.005
Male	39.5	60.5		
**School type**	**Yes (%)**	**No (%)**		
Public school	17.5	82.5	7.834	0.005
Private school	36.1	63.9		
**Nurse in the family**	**Yes (%)**	**No (%)**		
Yes	31.3	68.7	7.252	0.007
No	43.8	56.2		
**Mother’s education level**	**Yes (%)**	**No (%)**		
Illiterate	33.6	66.4	17.651	0.007
Primary school	30.6	69.4		
Secondary school	12.1	87.9		
High school	36.4	63.6		
Bachelor’s degree	41.9	58.1		
Postgraduate	45.1	54.9		
Unknown	18.2	81.8		
**Father’s education level**	**Yes (%)**	**No (%)**		
Illiterate	33.3	66.7	22.064	0.001
Primary school	41.7	58.3		
Secondary school	46.0	54.0		
High school	37.5	62.5		
Bachelor’s degree	26.1	73.9		
Postgraduate	17.5	82.5		
Unknown	22.2	77.8		
**Source of information about nursing**	**Yes (%)**	**No (%)**		
TV series/films	45.8	54.2	14.429	0.013
Social media	39.7	60.3		
School/teachers	44.4	55.6		
Family/close relatives	34.6	65.4		
Hospital experience	31.7	68.3		
Internet/news websites	21.2	78.8		

Abbreviation: *p* < 0.05; X^2^: Chi-Square test.

**Table 5:** Reliability Analysis of the APNIS and Subscales.

APNIS and Subscales	α*
Professional image	0.81
Perception	0.94
Care and therapeutic role	0.86
Communication	0.83
Informative role	0.83
Healing environment	0.73
Total scale	0.96

Abbreviation: α* = Cronbach’s alpha reliability.

## Data Availability

The authors confirmed that all relevant data are included in the article. The data that support the findings of this study are available on request from the corresponding author. The data are not publicly available due to privacy or ethical restrictions.

## Supplementary Materials

The following supporting information can be downloaded at: https://www.mdpi.com/article/10.3390/healthcare14111483/s1, File S1: Ethic Approval.
