# Supplementary material for: Public Image of Nursing Among High School Adolescents in Türkiye: Implications for the Future Healthcare Workforce
Source: Healthcare (Basel). 2026 May 27;14(11):1483. doi: 10.3390/healthcare14111483 (PMC13256970; doi:10.3390/healthcare14111483)
Supplement: Supplementary file 1 [file healthcare-14-01483-s001.zip › healthcare-4228625-supplementary.pdf]

File S1 **ETHIC APPROVAL**

İSTANBUL GEDİK ÜNİVERSİTESİ - Sağlık Bilimleri Etik Kurul Komisyonu
Tarih:04/02/2026 10:13
Sayı: E-11470191-050.04-2026.173340.21
00000412398

T.C.
İSTANBUL GEDİK ÜNİVERSİTESİ
Sağlık Bilimleri Etik Kurul Komisyonu

Sayı : E-11470191-050.04-2026.173340.21 04.02.2026
Konu : Etik Kurul Başvurusu (Tuba Çatak, Filiz Coşkun, Derya Gündüz Hoşgör)

Sayın Dr. Öğr. Üyesi Tuba ÇATAK (Sorumlu Araştırmacı)
Sayın Filiz Coşkun , (Yardımcı Araştırmacı)
Sayın Derya Gündüz Hoşgör (Yardımcı Araştırmacı)

Üniversitemiz Sağlık Bilimleri Etik Kurul Komisyonunun 03.02.2026 tarihli ve 2026/2 sayılı toplantısında, 412381 barkod numaralı "Türkiye'deki Ergenler Tarafından Algılanan Hemşirelik İmajı ve Etkileyen Faktörlerin Belirlenmesi" adlı başvurunuz görüşüldü. Yapılan görüşme sonunda, 412381 barkod numaralı "Türkiye'deki Ergenler Tarafından Algılanan Hemşirelik İmajı ve Etkileyen Faktörlerin Belirlenmesi" adlı başvurunuzun etik olarak uygun olduğuna katılanların oy birliği ile karar verildi.

Prof. Dr. Birol ÖZKALP
Etik Kurul Başkanı

Bu belge güvenli elektronik imza ile imzalanmıştır.
Belge Doğrulama Kodu: 915E33FC-7952-401E-8FB8-A371A96F2617 Belge Doğrulama Adresi: https://www.turkiye.gov.tr/istanbul-gedik-universitesi-ebys

Adres: sağlık bilimleri fakültesi
Telefon No: 4001
Faks No: 0
e-Posta: info@gedik.edu.tr
KEP Adresi: gedikuniversitesi@hs01.kep.tr

Ayrıntılı bilgi için: Elin KARADAĞ YAVUZ
Sekreter
Telefon No: 4001

İSTANBUL GEDİK ÜNİVERSİTESİ - Sağlık Bilimleri Etik Kurul Komisyonu
Tarih:03/03/2026 13:13
Sayı: E-11470191-050.04-2026.173340.38
00000415663

T.C.
İSTANBUL GEDİK ÜNİVERSİTESİ
Sağlık Bilimleri Etik Kurul Komisyonu

Sayı : E-11470191-050.04-2026.173340.38 03.03.2026
Konu : Etik Kurul Başvurusu (Dr. Öğr. Üyesi Tuba ÇATAK, Dr. Ece UYSAL KASAP)

Sayın Dr. Öğr. Üyesi Tuba ÇATAK (Sorumlu Araştırmacı)
Sayın Dr. Ece UYSAL KASAP (Yardımcı Araştırmacı)

Üniversitemiz Sağlık Bilimleri Etik Kurul Komisyonunun 03.02.2026 tarihli ve 2026/2 sayılı toplantısında, 04.02.2026 tarih ve 2026-02 sayılı ile 412398 barkod numaralı karar ile etik onay alan "Türkiye'deki Ergenler Tarafından Algılanan Hemşirelik İmajı Ve Etkileyen Faktörlerin Belirlenmesi" adlı çalışmasıyla ilgili düzeltme talebini içeren 415379 barkod numaralı ek dilekçesi görüşüldü. Yapılan görüşme sonunda dilekçede yer alan "çalışmanın istatistiksel analizlerinin yürütülmesi, verilerinin yorumlanması, makalenin yazımı ve araştırmanın raporlanması dahil olmak üzere çalışmanın bilimsel süreçlerinin tüm aşamaları için Dr. Ece UYSAL KASAP'ın çalışmaya dâhil edilmesinin etik olarak onaylanması" değerlendirme neticesinde etik olarak uygun olduğuna katılanların oy birliği ile karar verildi.

Prof. Dr. Birol ÖZKALP
Etik Kurul Başkanı

Bu belge güvenli elektronik imza ile imzalanmıştır.
Belge Doğrulama Kodu: 8B761E0D-780E-4272-B08D-D2F2787C2475 Belge Doğrulama Adresi: https://www.turkiye.gov.tr/istanbul-gedik-universitesi-ebys
Adres: sağlık bilimleri fakültesi
Telefon No: 4001
Faks No: 0
e-Posta: info@gedik.edu.tr
KEP Adresi: gedikuniversitesi@hs01.kep.tr

Ayrıntılı bilgi için: Elin KARADAĞ YAVUZ
Sekreter
Telefon No: 4001
